# Near-Wall Settling Behavior of a Particle in Stratified Fluids

**DOI:** 10.3390/mi13122070

**Published:** 2022-11-25

**Authors:** Minglu Dai, Chengxu Tu, Pengfei Du, Zhongke Kuang, Jiaming Shan, Xu Wang, Fubing Bao

**Affiliations:** Zhejiang Provincial Key Laboratory of Flow Measurement Technology, China Jiliang University, Hangzhou 310018, China

**Keywords:** particle sedimentation, near-wall sedimentation, stratified fluid, shear-thinning fluid

## Abstract

The phenomenon of near-wall particle settling in a stratified fluid is an emerging topic in the field of multiphase flow, and it is also widely found in nature and engineering applications. In stratified fluids, particle settling characteristics are affected by the physical and chemical properties of the upper and lower fluids, the particle size, the particle density, and the initial sedimentation conditions. In this study, the main objective is to determine the effect of liquid viscosity and particle density on the detaching process, and the trajectory and velocity of near-wall settling particles in stratified fluids. The inertia and velocity of the particle had a greater impact on the tail pinch-off model in low-viscosity lower fluids; that is, the lower the inertia and velocity, the more apparent the order between deep and shallow seal pinch-off. In comparison, in high-viscosity lower fluids, the tail pinch-off models of different inertia and velocity particles were similar. In terms of particle trajectory, the transverse motion of the particle in the low-viscosity lower fluid exhibited abrupt changes; that is, the particles moved away from the wall suddenly, whereas in the high-viscosity lower fluid, the transverse movement was gradual. Due to the existence of the wall, the transverse motion direction of the free settling particles in the stratified fluid, which is determined by the rotation direction of the particles, changed to a direction away from the wall regardless of the particle rotation direction. This transverse movement also caused the particle settling velocity to drop suddenly or its rising rate to decrease, this is because part of the energy was used for transverse motion and to increase the transverse velocity. In our study, the near-wall settling of particles in a stratified fluid mainly affected the particle trajectory; that is, forced movement away from the wall, thus changing the particle velocity. This characteristic provides a new approach to manipulate particles away from the wall.

## 1. Introduction

The sedimentation of particles is a universal physical phenomenon in natural and industrial processes; examples include inhaled particles in the respiratory tract [1], sediment deposition and resuspension in rivers [2], flow of blood [3], and air pollution [4]. The settling trajectory and velocity of spherical particles have been widely studied [5,6,7,8]. However, in industrial processes, particles take diverse shapes, and sedimentation modes include vertical sedimentation, horizontal sedimentation, and oscillating [9]. The particle settling characteristics are not only affected by the shape and material of the particle, but also by the fluid properties. In particular, in some cases, the particle needs to settle through the stratified fluid layers, such as thermoclines, haloclines, and estuaries, with large gradients in fluid density [10]. A rigid sphere can either maintain an equilibrium at the liquid–liquid interface or pass through the interface. Maru et al. [11] proposed a model that can predict the critical radius of a sphere. When the radius of a sphere is less than the critical radius, the sphere cannot pass through the interface. In different stratified fluids, because of the differences in the viscosity and interfacial tension between fluids, the modes whereby particles pass through the interface can be divided into two types: (i) film drainage mode: as the particle approaches the interface, a film is formed between the particle and the lower fluid, and the film drains gradually [12,13]; (ii) tailing mode: when the particle passes through the interface, the particle is still being enveloped by the upper fluid, which is like a tail behind the particle [10,14]. In the tailing mode, tail pinch-off occurs as a result of the opposing flow directions in the tail according to Dietrich et al. [15]. Based on their particle image velocimetry (PIV) results obtained, the fluid in the upper part of the tail flows upward under the effect of interface tension and buoyancy, and the fluid near the particle in the lower part of the tail flows downward. Notably, the pinch-off modes and tailing configurations are different in stratified fluids of varying viscosity. Pierson et al. [14,16] used four viscosity ratios to study the settling characteristics of particles, and established a simple model for each condition, and the model was validated based on experimental results. The drag was found to increase when the particle passed through the interface because of the buoyancy of the upper fluid surrounding the particle [17]. Yick et al. [17] also pointed out that the region of the upper fluid surrounding the particle whose size is the power of one half of the ratio of the viscosity of the moving fluid and the buoyancy frequency will increase the drag. However, in industrial processes, not all particles settle freely, and thus many studies have focused on the settling process near a wall [18,19,20].

The end effects have been noted as early as 1963 [21], and in 1967, Goldman et al. studied the motion of a sphere parallel to a wall [22]. Due to the wall effect, the particle is not only closer or far away from the wall, but also rotated, and the rotation direction is related to the fluid properties [23]. Similarly, Singh et al. [20] pointed out that a sphere settling near a wall in a Newtonian fluid rotates abnormally and drifts away from the wall to a stable position. However, it moves toward the wall in a shear thinning fluid, whereas a settling cylinder moves away from the wall on the plane perpendicular to the wall. This shows that the interaction between the wall and sphere in a viscoelastic fluid is three-dimensional [20], which has been confirmed by Harrison et al. based on the 3D PIV technique. The flow field at the box center is two dimensional, while that near the wall of the box exhibits evident out-of-plane motion. Harrison et al. [24] also studied the effect of viscosity and elasticity on the near-wall particle settling characteristics. Their results showed that a motion perpendicular to the wall is evident in water and shear-thinning fluids, whereas it is not significant in a Boger fluid [25], and the transverse migration velocity decreases with increasing distance between the wall and the particle, regardless of the fluid properties [26].

In this study, the near-wall sedimentation characteristics of a single particle in a stratified fluid were studied. This study focuses on the effects of wall distance, particle density and fluid viscoelasticity on particle settlement in near-wall stratified fluid.

## 2. Experimental Design

**Figure 1:**
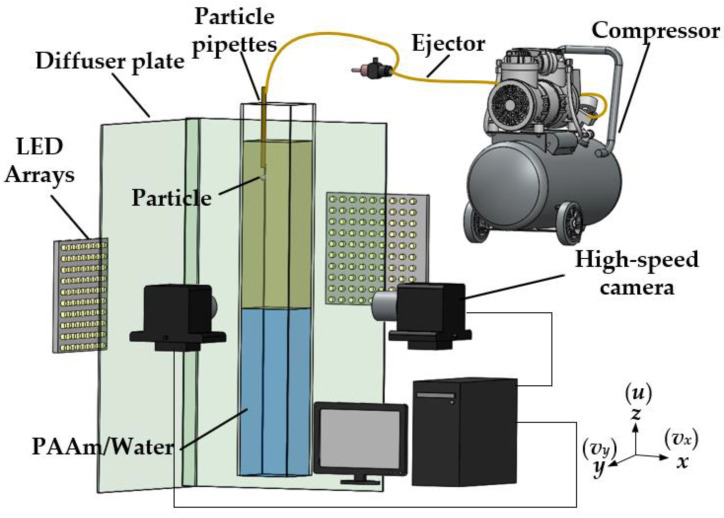
Experimental setup for near-wall particle settling in a stratified fluid based on a high-speed shadowgraph imaging.

Figure 1 shows the experimental setup used to trace the particle motion in stratified fluids. In this study, experiments were conducted in an acrylic tank (150 mm × 150 mm × 1000 mm) filled with a stratified fluid. By using an ejector to generate vacuum pressure on the particle, the particle could be released without perturbation. Two high-speed cameras (FASTCAM Mini UX, Photron, Yonezawa, Japan) were used to facilitate high-speed shadowgraph binocular-imaging and to record the particle trajectory in *x*–*z* and *y*–*z* planes. The light source was provided by two LED arrays, and the binocular-imaging system was used to track the particle motion.

**Figure 2:**
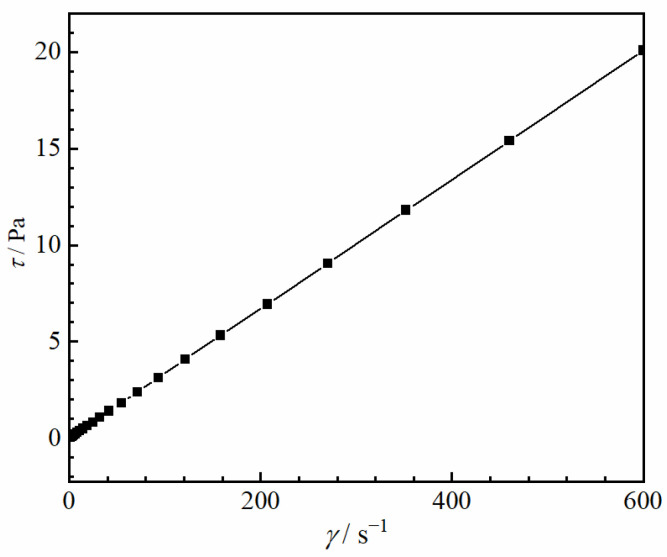
Rheograms of mineral oil.

**Figure 3:**
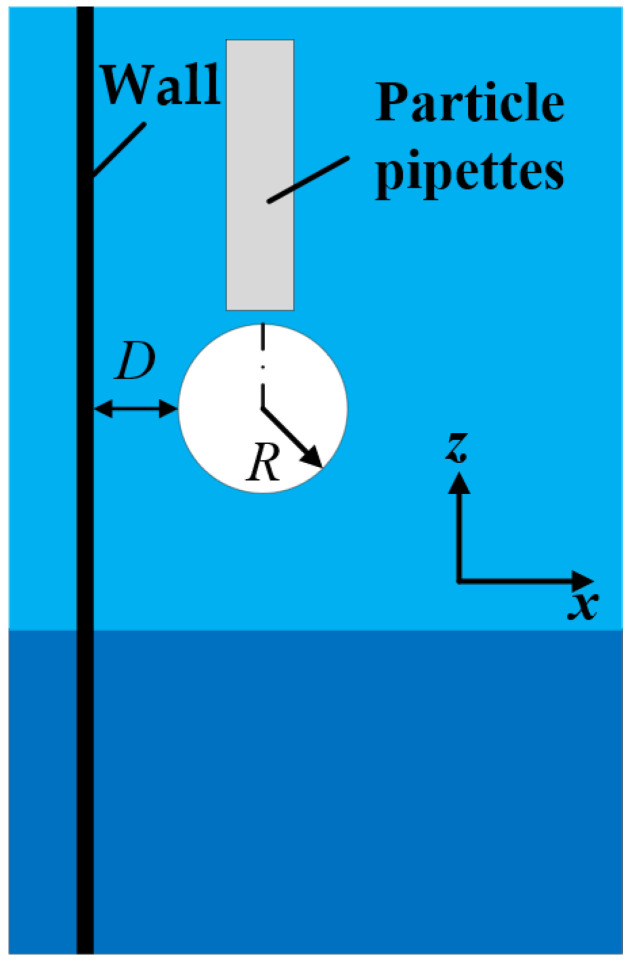
Schematic of the particle release position and the gap *D* between the particle and wall.

The stratified fluids used in the experiments were as follows: (I) a stratified fluid with a Newtonian–Newtonian fluid interface comprising mineral oil (Sigma-Aldrich M5904, Shanghai, China) as the upper fluid and water as the lower fluid; (II) a stratified fluid with a Newtonian–non-Newtonian fluid interface comprising oil as the upper fluid and 0.5 wt% polyacrylamide (PAAm, Shanghai Macklin Biochemical Co., Shanghai, China) as the lower fluid. The densities of the water, oil, and PAAm were 1000, 840, and 1005 kg/m^3^, respectively. Accordingly, oil could be used as the upper fluid. Figure 2 shows the rheological characteristics of the mineral oil, and the rheological properties of water and PAAm refer to our previous research [27]. The data were measured at 20 °C using a rheometer (MCR 302 Multidrive, Anton Paar Co., Graz, Autria). Based on the measurement results, the shear stress *τ* was linearly correlated with the shear rate *γ* of the water and mineral oil, whereas the same relationship in the case of the PAAm solution was nonlinear. Since the viscosity *μ* is the ratio of *τ* to *γ*, and the rheological curve slope for the PAAm solution gradually decreases, the viscosity of the PAAm solution decreases with the increase in *γ*. Thus, the PAAm solution is a shear-thinning fluid. The storage modulus and loss modulus data of PAAm solution in our previous study demonstrates that the PAAm solution is a viscoelastic fluid [27]. Two types of particles were selected for the experiments (Table 1), and the radius of both these particles *R* was set to 2.5 mm, to eliminate the influence of the geometry; only the densities and type of material differed. The materials of the particles used in the experiment were polytetrafluoroethylene (PTFE) and silicon nitride ceramics (Si_3_N_4_). The diameter variation of the particles was less than 0.25 μm. The dimensionless distance D* was adopted to describe the particle size and the distance *D* between the particle and the wall (Figure 3):

(1) D*=D/R

## 3. Results and Discussion

### 3.1. Force Analysis

**Figure 4:**
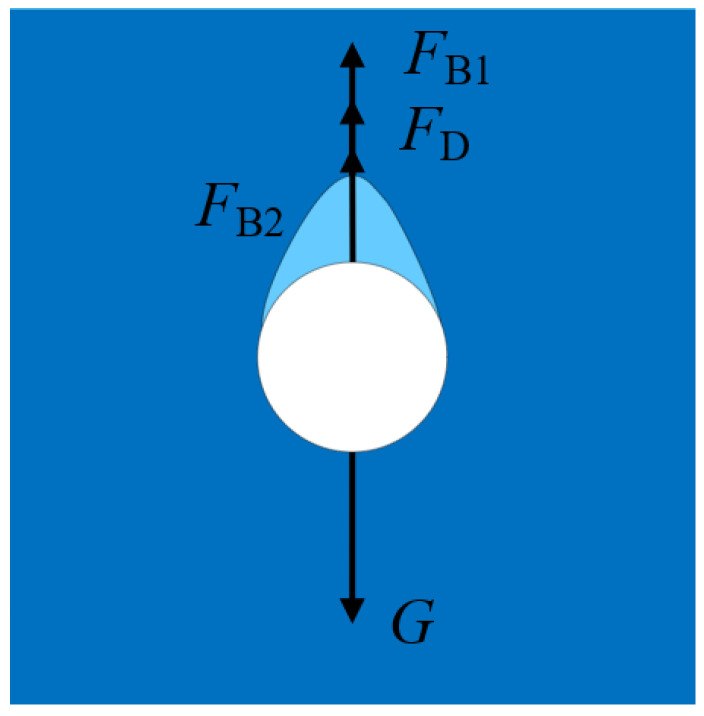
Force diagram of a particle in the lower fluid.

After a particle detaches from a liquid–liquid interface, a droplet of the upper fluid will attach to the tail of the particle. Thus, the forces acting on the particle include the gravity *G*, particle buoyancy *F*_B1_, attached droplet buoyancy *F*_B2_, and total drag force *F*_D_ (Figure 4):

(2) $$G=F_{D}+F_{B1}+F_{B2}$$

(3) $$G=\frac{4}{3}\pi R^{3}\rho_{s}g$$

(4) $$F_{B1}=\frac{4}{3}\pi R^{3}\rho_{l}g$$

(5) $$F_{B2}=V_{d}\rho_{l}g$$

where *ρ*_s_ is the density of the particle, *ρ*_l_ is the density of the lower fluid, and *V*_d_ is the volume of the attached droplet. The total drag force of the settling particle in a Newtonian fluid has been widely studied and can be confirmed by the Reynolds number (*Re* = 2*ρ*_l_*u*_z_*R*/*μ*). However, it becomes more complicated in the PAAm solution. Herein, the expression for the total drag force in a power fluid was used [27,28]:

(6) $$F_{D}=12\pi kR^{2}{\left(\frac{u_{z}}{2R}\right)}^{n}f\left(n\right)$$

where *k* is the consistency index, *n* is the power law index, and *f*(*n*) is a function of the power law constant. Based on the power law model [29], $\tau =k\gamma^{n}$, *k* = 0.572, and *n* = 0.484 by fitting the data [27]. The Reynolds number for a PAAm solution is also related to *k* and *n*, and is given by [30]:

(7) $$Re^{*}=\frac{{\left(2R\right)}^{n}u_{z}^{2-n}\rho_{l}}{k}$$

As for the Newtonian fluid, the expression for the total drag force is:

(8) $$F_{D}=C_{D}\frac{\rho u_{z}^{2}\pi R^{2}}{2}$$

where *C*_D_ is the drag coefficient and the expression is *C*_D_ = 0.28 + 6/*Re*^1/2^ + 21/*Re*.

Due to the wall effect slightly influencing the terminal settling velocity, the mean values of different D* are used to calculate *Re* and Re*. The mean terminal settling velocities were 0.298 m/s (for the PTFE particles in an oil–water stratified fluid), 0.643 m/s (for Si_3_N_4_ particles in an oil–water stratified fluid), 0.047 m/s (for PTFE particles in an oil–PAAm stratified fluid), and 0.123 m/s (for Si_3_N_4_ particles in an oil–PAAm stratified fluid). Therefore, the *Re* values for the oil–water stratified fluid were 1678 (for PTFE particles) and 3614 (for Si_3_N_4_ particles), respectively, and the Re* values for the oil–PAAm stratified fluid were 1.3 (for PTFE particles) and 5.7 (for Si_3_N_4_ particles), respectively.

### 3.2. Tail and Particle Trajectory

**Figure 5:**
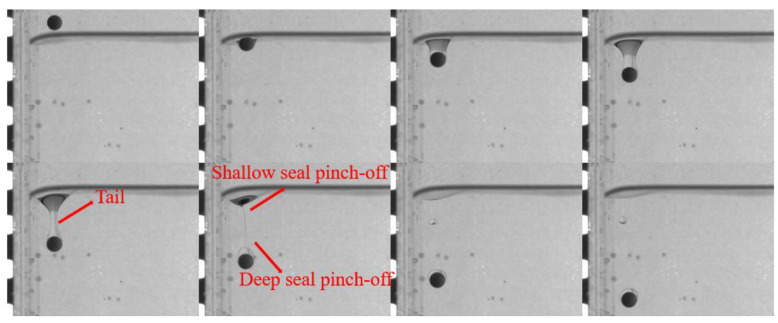
Pinch-off process of PTFE particles in an oil–water stratified fluid. The dimensionless distance D* = 1, and the time interval Δ*t* between adjacent images is 25 ms.

**Figure 6:**
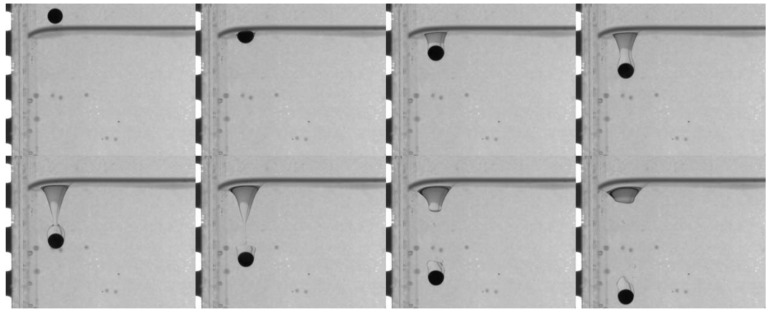
Pinch-off process of Si_3_N_4_ particles in an oil–water stratified fluid. The dimensionless distance D* = 1, and the time interval Δ*t* between adjacent images is 15 ms.

In this section, the characteristics of different particles detaching from different liquid–liquid interfaces are studied. Figure 5 and Figure 6 show the detachment processes of the PTFE and Si_3_N_4_ particles at the oil–water interface, respectively. It has been found that the flow in the lower part of the tail is downward, while it is upward in the upper part; this is the reason for the pinch-off phenomenon [15]. The pinch-off model is also related to the particle and fluid properties. Considering the dimensionless distance D* and the fact that the particle geometry conditions are consistent as shown in Figure 5 and Figure 6, the particle inertia and particle-to-fluid density are the main factors affecting the tail pinch-off model. The multimodal tail pinch-off process is the result of competing buoyancy, surface tension, viscous forces, inertia, gravity, and particle rotation. When the particle reaches the interface, the settling velocity decreases rapidly, and the particle accelerates immediately, promoting a deep seal pinch-off [10]. Due to the lower particle-to-fluid density compared with that of the Si_3_N_4_ particle, the inertia provided by the PTFE particles was insufficient to support the downward motion of the tail near the particle. Meanwhile, the interface should be rebounded, and the tail near the interface recedes upward. In addition, the PTFE particles have a lower settling velocity than the Si_3_N_4_ particles, which means that the PTFE particles require a longer duration to fall to the same height, giving the upper part of the tail more time to recede. Therefore, the pinch-off of the tail occurs twice. After the completion of deep seal pinch-off, a shallow seal pinch-off occurs. Finally, the tail is transformed into a satellite droplet. Similar to the PTFE particles, the acceleration process results in a deep seal pinch-off of the tail of the Si_3_N_4_ particle. However, because of the higher mass, settling velocity, and inertia, the tail close to the particle is elongated, easily inducing capillary instability [14]. As soon as pinch-off occurs, the instability propagates upward, and a series of droplets are generated.

**Figure 7:**
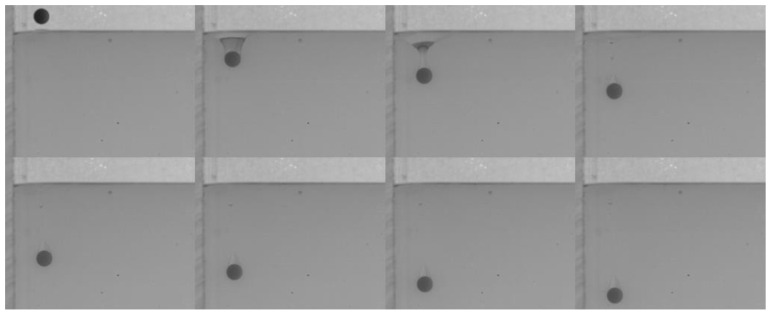
Pinch-off process of PTFE particles in an oil–PAAm stratified fluid. The dimensionless distance D* = 1, and the time interval Δ*t* between adjacent images is 6.5 ms.

**Figure 8:**
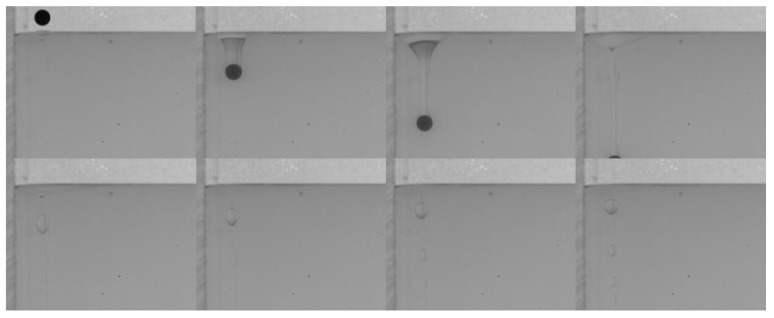
Pinch-off process of Si_3_N_4_ particles in an oil–PAAm stratified fluid. The dimensionless distance D* = 1, and the time interval Δ*t* between adjacent images is 6.5 ms.

**Figure 9:**
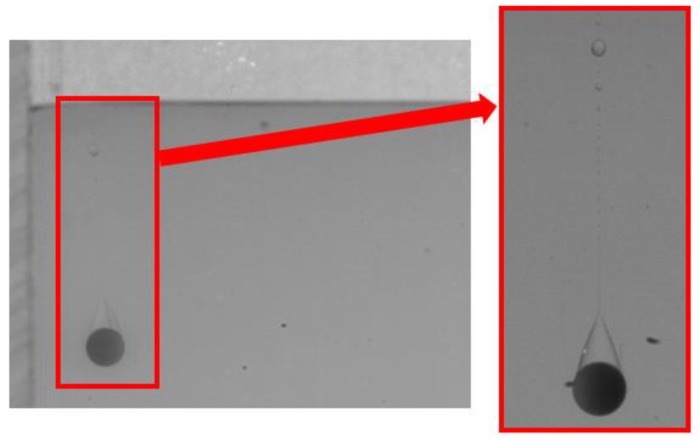
Local enlarged image of the PTFE particle tail detaching from the oil–PAAm interface.

When the lower fluid is 0.5 wt% polyacrylamide solution, the tail pinch-off model is similar for both PTFE and Si_3_N_4_ particles. The shallow and deep seal pinch-off phenomena occur almost simultaneously, and then series satellite droplets are formed (Figure 7 and Figure 8). Unlike the deep seal pinch-off characteristics in the oil–water stratified fluid in which the pinch-off location is near the particle, the locations of both deep and shallow seal pinch-off in the oil–PAAm stratified fluid are close the interface. This can be attributed to the shear-thinning behavior of the PAAm solution. Once the particle enters the PAAm solution, its settling velocity decreases rapidly, but then plateaus. The decreasing shear velocity indicates that the viscosity of the PAAm solution near the interface is the lowest, favoring the pinch-off. Thus, both shallow and deep seal pinch-off phenomena occur near the interface. After the pinch-off, the upper half of the tail recedes toward the interface, during which the smaller satellite droplets detach from the tail. This phenomenon also occurs in the lower half of the tail and is more evident therein. In Figure 9, a series of satellite droplets detach along a straight line due to the propagation wave, which is similar to the shedding of satellite bubbles behind the air cavity behind the impact of the particle [31,32].

**Figure 10:**
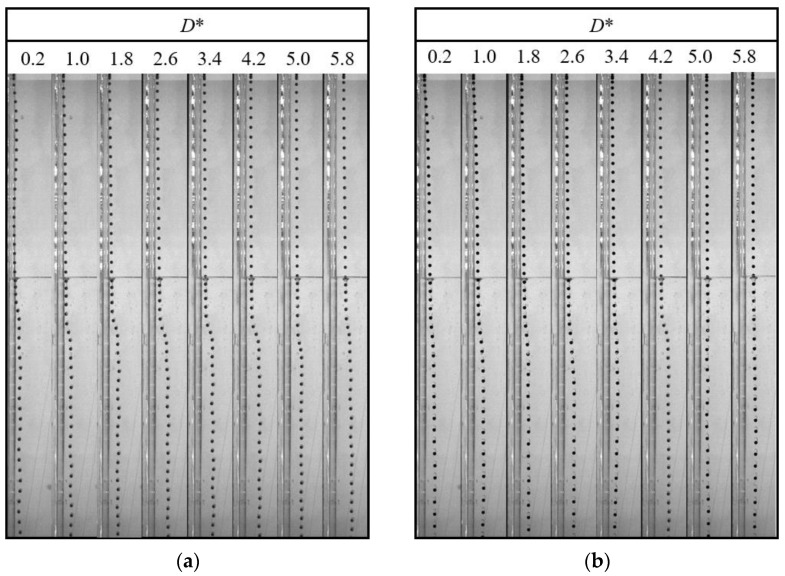
Particle trajectory in an oil–water stratified fluid at different dimensionless distances D*: (**a**) PTFE particle with Δ*t* = 50 ms; (**b**) Si_3_N_4_ particle with Δ*t* = 30 ms.

**Figure 11:**
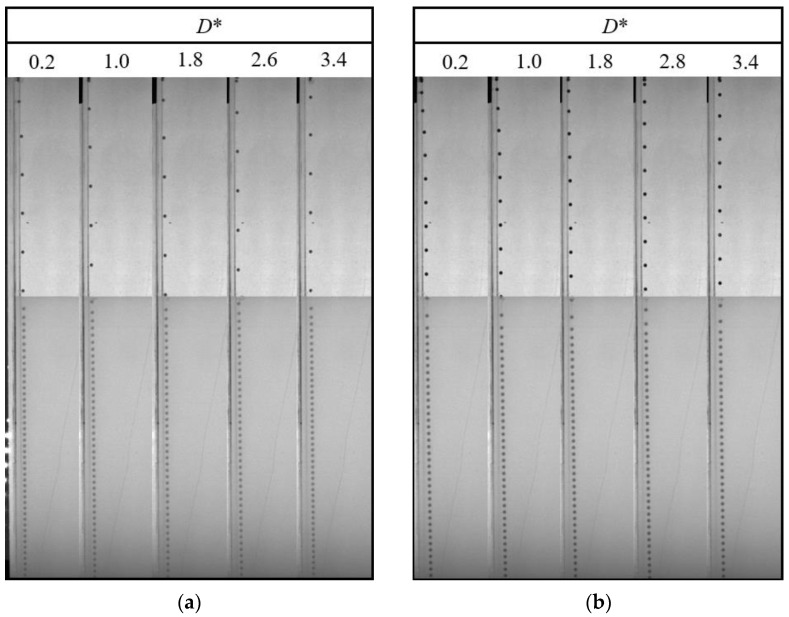
Particle trajectory in an oil–PAAm stratified fluid at different dimensionless distances D*: (**a**) PTFE particle with Δ*t* = 50 ms; (**b**) Si_3_N_4_ particle with Δ*t* = 20 ms.

Many studies have been conducted on the near-wall settling and rising characteristics of particles or bubbles [33,34,35,36]. Both experimental and simulation results have shown an anomalous rolling of the particle during near-wall settling regardless of the type of fluid (Newtonian or non-Newtonian fluid) [18,19,37]. In a stratified fluid, particle motion in the upper fluid depends on the physical properties of the fluid and the particle. However, after the particle enters the lower fluid, an attached droplet that attaches to the tail of the particle will influence the wake of the particle, and it is unknown whether the attached droplet influences the trajectory [10]. As shown in Figure 10, there is a clear sudden transverse migration of the trajectory after the pinch-off occurs, particularly for high D* values of the lower fluid. This is consistent with previous studies which attributed this phenomenon to unbalanced vortices [18,37,38]. Compared with the particle settling trajectory in water, the particle with an attached droplet does not seem to affect the overall trend but only changes the transverse displacement. In addition, Figure 10a,b demonstrate that the wall has less effect on the Si_3_N_4_ particle as the force induced by the unbalanced vortex cannot significantly push the particle because of its larger mass and related greater inertia. The displacement of transverse migration first increases and then decreases with the increase of D*. Due to the lower viscosity of the water, the settling trajectory of the particle with an attached droplet is more affected by the inertia than the viscosity. This is not the case in the oil–PAAm stratified fluid. Both the PTFE and Si_3_N_4_ particles migrate away from the wall gradually and slightly (Figure 11), which is the result of the combined influence of the higher fluid’s viscosity and attached droplet. Different from the transverse migration of particles in an oil–water stratified fluid, the transverse migration of particles in an oil–PAAm stratified fluid decreases with the increase of D* after settling to the bottom. Although the PAAm solution is a shear-thinning fluid, its viscosity is always greater than that of water [27]. Moreover, the settling velocities of these two types of particles are lower in an oil–PAAm stratified fluid, which is shown in Section 3.3. Hence, the fluid viscosity of the oil–PAAm stratified fluid has a greater effect on the trajectory. In Figure 9, the shape of the attached droplet is streamlined, which is analogous to the air cavity behind particle [31,32,39], but the tip of attached droplet is sharp and this sharp tip is typical of the presence of a negative wake [7]. A streamlined attached droplet or cavity can reduce the number of wake vortices. As mentioned above, the anomalous rolling of the particle is due to unbalanced vortices. Overall, a streamlined shape of the attached droplet favors particle settling along an inclined straight line. In comparison, the transverse migration of the trajectory in the *y*–*z* plane is more random, and the transverse migration direction depends on the direction of rotation of the particle. The presence of the wall pushes the particle away from the wall regardless of the direction of rotation of the particle. When there is no wall, the particle trajectory and the particle rotation direction are related to the wake vortex [5,40]. When there is a wall, according to Bernoulli’s principle, because the wall is non-slip, the fluid between the particle and the wall has lower velocity and higher pressure than that of the fluid under free shear. Thus, the particle will be pushed away from the wall.

### 3.3. Velocity

**Figure 12:**
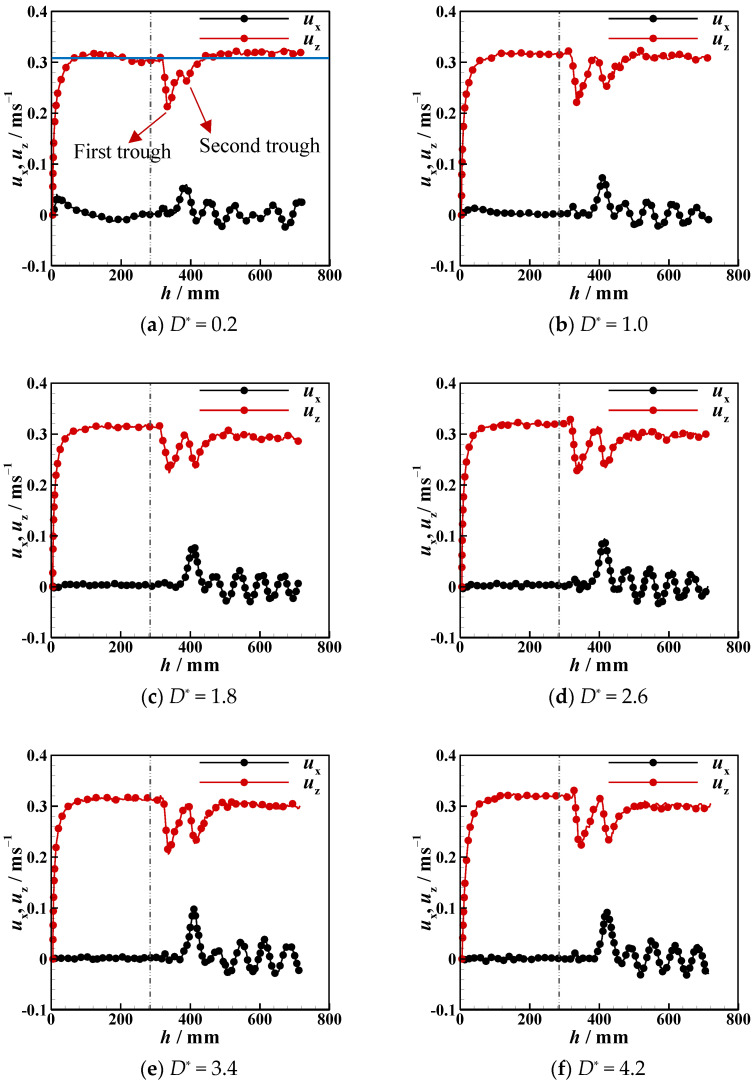
Near-wall settling velocity and transverse velocity of PTFE particles in oil–water stratified fluid at different dimensionless distances D*.

**Figure 13:**
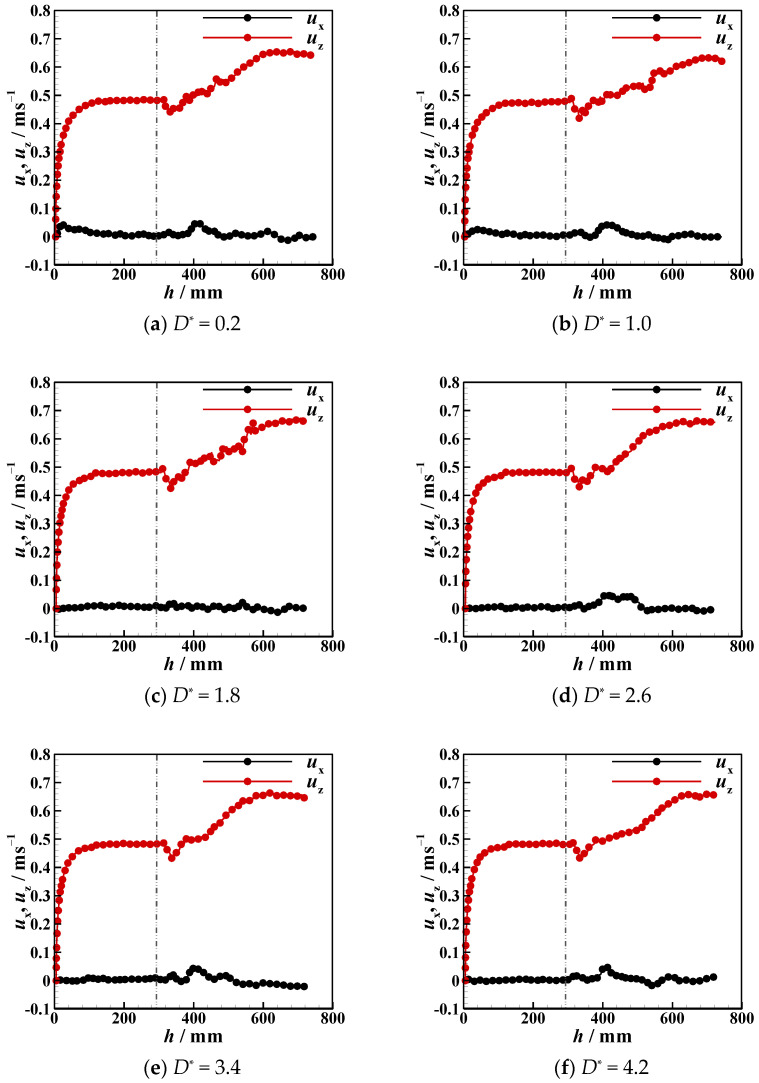
Near-wall settling velocity and transverse velocity of Si_3_N_4_ particles in oil–water stratified fluid at different dimensionless distances D*.

**Figure 14:**
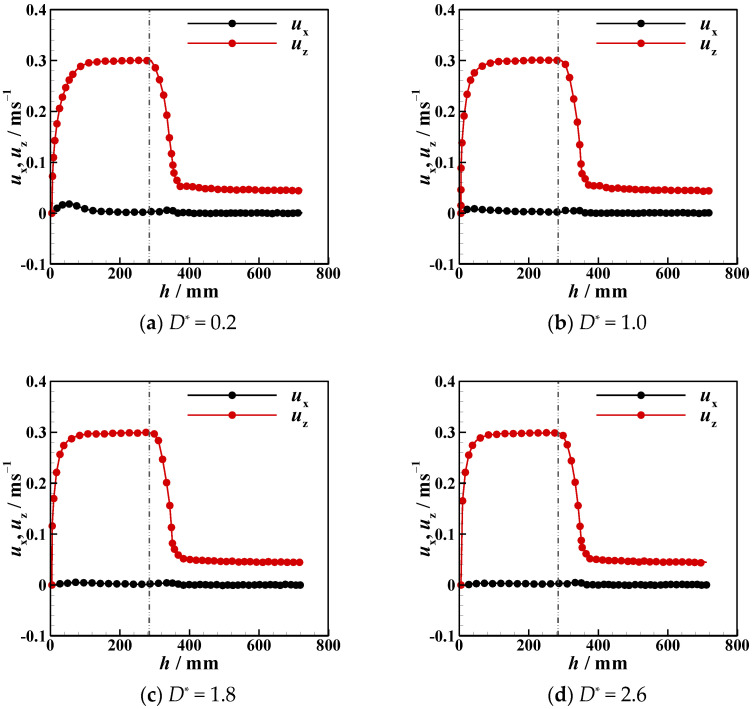
Near-wall settling velocity and transverse velocity of PTFE particles in oil–PAAm stratified fluid at different dimensionless distances D*.

**Figure 15:**
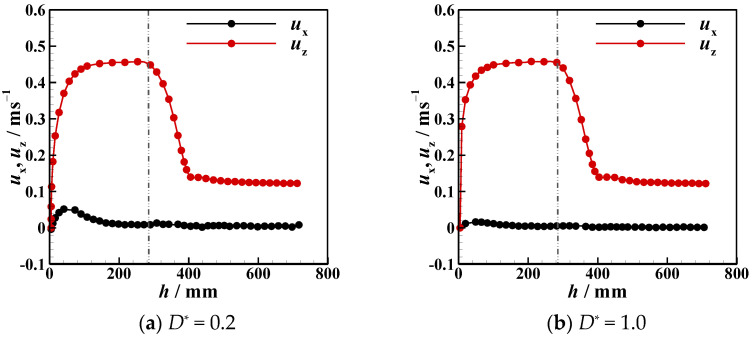

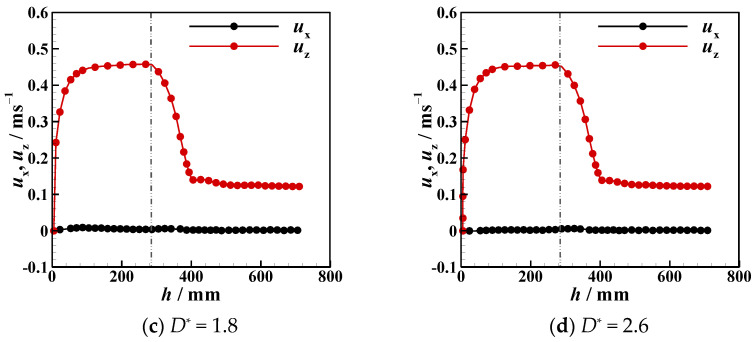
Near-wall settling velocity and transverse velocity of Si_3_N_4._ particles in oil–PAAm stratified fluid at different dimensionless distances D*.

**Figure 16:**
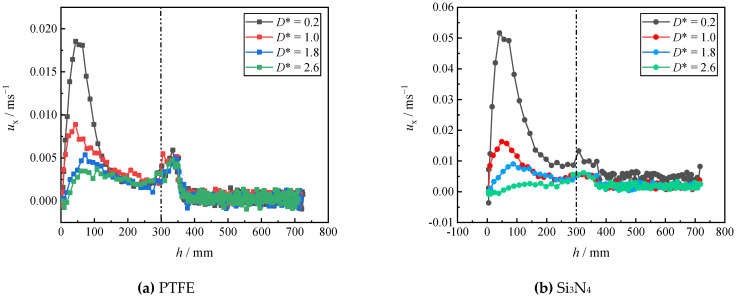
Near-wall transverse velocity of PTFE and Si_3_N_4_ particles in oil–PAAm stratified fluid at different dimensionless distances D*.

**Figure 17:**
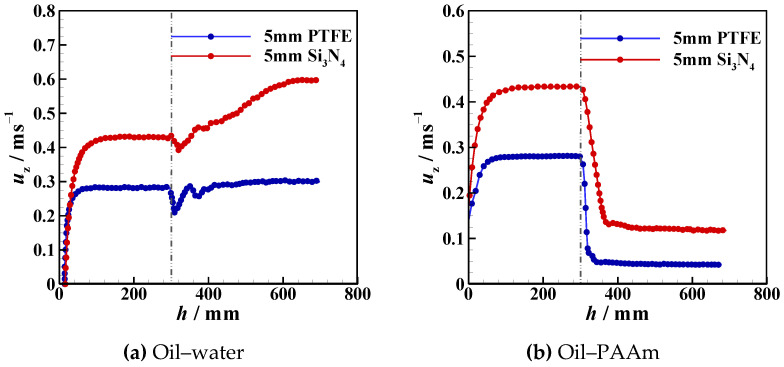
Free settling velocities of PTFE and Si_3_N_4_ particles in oil–water and oil–PAAm stratified fluids.

Due to the presence of an interface, the settling velocity is related to the process whereby the particle detaches from the liquid–liquid interfaces, the tail pinch-off mechanism, and the transverse migration. In this section, the settling velocity (*u*_z_) of different particles in different stratified fluids is discussed. In Figure 12, Figure 13, Figure 14, Figure 15, Figure 16 and Figure 17, the left side of the dotted line represents the particle velocity in the upper fluid, and the right side of the dotted line represents the particle velocity in the lower fluid. After the particle is released, *u*_z_ rises quickly, and the particle reaches the force balance state and starts to move uniformly in the upper fluid, which is common for both PTFE and Si_3_N_4_ particles in oil–water and oil–PAAm stratified fluids. When the lighter PTFE particle arrives at the oil–water interface, its kinetic energy is transferred to the surrounding fluid, and *u*_z_ rapidly decreases to the first trough before pinch-off occurs (Figure 12). Due to the particles used in the experiment, even the lighter PTFE particles cannot stay in the liquid–liquid interface, the particles enter the lower fluid before film drainage is completed. At this time, the front part of the particle is still wrapped in mineral oil while the pinch-off of the tail behind the particle has not taken place. Thus, the whole particle is wrapped in mineral oil. Therefore, the particles are in the slip boundary condition, and the friction drag of the particles will be reduced [41]. Thus, this velocity decreasing process is the result of the competition between the particle inertia, gravity, buoyancy, friction drag, form drag, and interface tension [10]. Subsequently, the pinch-off occurs, and the pull force applied by the interface on the particle vanishes, and *u*_z_ starts to increase. As mentioned above, the particle trajectory in the oil–water stratified fluid has a sudden transverse migration in the lower fluid, which is also reflected in both vertical and transverse velocity (*u*_x_) variations. The appearance of transverse migration means an increase in *u*_x_. When transverse migration occurs, *u*_z_ drops to the second trough, while *u*_x_ reaches its maximum value. Considering the energy conservation principle, a rising *u*_x_ implies a decreasing *u*_z_. With increasing D*, the value of the second trough further declines, which corresponds to the gradually evident transverse migration mentioned in Section 3.2. After the sudden transverse migration distancing the particle and the wall, the wake vortex and the forces become balanced, and *u*_z_ of the particle plateaus again. Regardless of D*, the value of *u*_z_ in oil with higher viscosity or in water with lower viscosity is approximately 0.3 m/s. This is because the attached oil droplet provides buoyancy for the particle, and thus, *u*_z_ of the particle in water is close to that of the particle in oil. For *u*_x_, it fluctuates with a small amplitude because of periodic and alternate shedding vortices [37,38]. As for the Si_3_N_4_ particle, the mechanism of the first trough is similar to that exhibited by the PTFE particle due to the particle detaching motion. Conversely, the transverse migration of the Si_3_N_4_ particle trajectory does not occur as abruptly as that of the PTFE particle. Therefore, the effect of transverse migration on *u*_z_ mainly causes the slowing down of the rising rate of *u*_z_. In Figure 13, particularly Figure 13d,e, there is a transient plateau of *u*_z_. Furthermore, the trend of *u*_x_ of the Si_3_N_4_ particle has a smaller change rate than that of the PTFE particle, confirming the weaker influence of the wake on the particle with greater inertia. Furthermore, the terminal vertical velocity in water is greater than the velocity in oil, indicating that the buoyance provided by the attached droplet is limited for the Si_3_N_4_ particle compared with the PTFE particle. Owing to the high viscosity of the PAAm solution, *u*_z_ rapidly reaches plateau after the plummeting of *u*_z_, and the terminal velocity is significantly lower than that in oil (Figure 14 and Figure 15). The wall still has an effect on *u*_x_, even though the overall from the inclined straight trajectory in the oil–PAAm stratified fluid, *u*_x_ is very small compared with *u*_x_ in oil–water stratified fluid. Owing to the low Re* (1.3 for the PTFE particle and 5.7 for the Si_3_N_4_ particle), the particle wake is very stable, and both *u*_z_ and *u*_x_ are nearly constant. In Figure 16, especially when the particle is near the wall, the pressure gradient between the particle and wall is large. So the wall changes the lift force of the particle, and the particle will migrate laterally [19]. Figure 16b, the smaller D* is, the greater the variation of *u*_x_ of particles; that is, the closer the particles are to the wall, the greater the force exerted by the wall on transverse migration of particles. Figure 17 shows that the velocity evolution in the absence of a wall is largely the same as the trend in the near-wall settling velocities. Furthermore, the free settling velocity is also close to the near-wall settling velocities. Both the trends and values of the velocity further demonstrate that the wall mainly influences the settling trajectory and transverse migration.

## 4. Conclusions

The trajectory, tail, and velocity of near-wall particles settling in different stratified fluids were studied. Due to the wall, the tail behind the particle was asymmetric before the occurrence of pinch-off. The tail pinch-off model differed for different particles and fluids. In the low-viscosity fluid, the velocity and inertia of the particle significantly influenced the tail pinching mode. In the oil–water stratified fluid, the low settling velocity and inertia of the PTFE particle led to an apparent asynchronous between deep and shallow seal pinch-off. When the velocity and inertia were sufficiently high by the time the heavier Si_3_N_4_ particle reached the liquid–liquid interface, the tail near the particle was elongated, and after the deep seal pinch-off occurred, satellite droplets were formed from the tail. Compared with that observed in water, the tail pinch-off model was less affected by the particle velocity and inertia when the lower fluid was the more viscous PAAm solution. Under this condition, the shallow and deep seal pinch-off occurred almost simultaneously for both the PTFE and Si_3_N_4_ particles, and satellite droplets were generated both from the part that was attached to the interface and from the tail of the attached droplet. In addition, under the influence of the wall, the near-wall particle always moved away from the wall after passing through the liquid–liquid interface, while the deflection direction was determined by the rotation direction of the particles in the free settling process. The particles motion direction turns suddenly when the viscosity of the lower fluid was low (the oil–water stratified fluid in this study), whereas the particles gradually moved away from the wall when the viscosity was high (the oil–PAAm stratified fluid in this study). The sudden transverse motion of the particles in the low-viscosity fluid also caused the particle settling velocity to decrease suddenly and the transverse velocity to reach its peak. The wall could force the particle to move away from it regardless of the rotation direction of the particle itself, though it did not seem to have an evident effect on the particle settling velocity or tail pinch-off model.

## Figures and Tables

**Table 1:** Properties of particles.

Material	Density (kg/m^3^)	Diameter (mm)
Polytetrafluoroethylene (PTFE)	2200	5.0
Silicon nitride ceramics (Si_3_N_4_)	3200	5.0

## Data Availability

The data that support the findings of this study are available from the corresponding author upon reasonable request.

## References

[1] Bernstein D.M. (2004). A review of the influence of particle size, puff volume, and inhalation pattern on the deposition of cigarette smoke particles in the respiratory tract. Inhal. Toxicol..

[2] Kuehl S.A., Nittrouer C.A., Allison M.A., Faria L.E.C., Dukat D.A., Jaeger J.M., Pacioni T.D., Figueiredo A.G., Underkoffler E.C. (1996). Sediment deposition, accumulation, and seabed dynamics in an energetic fine-grained coastal environment. Cont. Shelf Res..

[3] Leslie H.A., Van Velzen M.J., Brandsma S.H., Vethaak A.D., Garcia-Vallejo J.J., Lamoree M.H. (2022). Discovery and quantification of plastic particle pollution in human blood. Environ. Int..

[4] Fernando H., Lee S., Anderson J., Princevac M., Pardyjak E., Grossman-Clarke S. (2001). Urban fluid mechanics: Air circulation and contaminant dispersion in cities. Environ. Fluid Mech..

[5] Horowitz M., Williamson C. (2010). The effect of Reynolds number on the dynamics and wakes of freely rising and falling spheres. J. Fluid Mech..

[6] Magnaudet J. The forces acting on bubbles and rigid particles. Proceedings of the ASME Fluids Engineering Division Summer Meeting, FEDSM.

[7] Zenit R., Feng J. (2018). Hydrodynamic interactions among bubbles, drops, and particles in non-Newtonian liquids. Annu. Rev. Fluid Mech..

[8] Jenny M., Dušek J., Bouchet G. (2004). Instabilities and transition of a sphere falling or ascending freely in a Newtonian fluid. J. Fluid Mech..

[9] Xia Z., Connington K.W., Rapaka S., Yue P., Feng J.J., Chen S. (2009). Flow patterns in the sedimentation of an elliptical particle. J. Fluid Mech..

[10] Magnaudet J., Mercier M.J. (2020). Particles, drops, and bubbles moving across sharp interfaces and stratified layers. Annu. Rev. Fluid Mech..

[11] Maru H., Wasan D., Kintner R. (1971). Behavior of a rigid sphere at a liquid—Liquid interface. Chem. Eng. Sci..

[12] Smith P., Van de Ven T. (1984). The effect of gravity on the drainage of a thin liquid film between a solid sphere and a liquid/fluid interface. J. Colloid Interface Sci..

[13] Jones A., Wilson S. (1978). The film drainage problem in droplet coalescence. J. Fluid Mech..

[14] Pierson J.-L., Magnaudet J. (2018). Inertial settling of a sphere through an interface. Part 2. Sphere and tail dynamics. J. Fluid Mech..

[15] Dietrich N., Poncin S., Li H.Z. (2011). Dynamical deformation of a flat liquid–liquid interface. Exp. Fluids.

[16] Pierson J.-L., Magnaudet J. (2018). Inertial settling of a sphere through an interface. Part 1. From sphere flotation to wake fragmentation. J. Fluid Mech..

[17] Yick K.Y., Torres C.R., Peacock T., Stocker R. (2009). Enhanced drag of a sphere settling in a stratified fluid at small Reynolds numbers. J. Fluid Mech..

[18] Liu Y.J., Nelson J., Feng J., Joseph D.D. (1993). Anomalous rolling of spheres down an inclined plane. J. Non-Newton. Fluid Mech..

[19] Becker L., McKinley G., Stone H. (1996). Sedimentation of a sphere near a plane wall: Weak non-Newtonian and inertial effects. J. Non-Newton. Fluid Mech..

[20] Singh P., Joseph D. (2000). Sedimentation of a sphere near a vertical wall in an Oldroyd-B fluid. J. Non-Newton. Fluid Mech..

[21] Tanner R. (1963). End effects in falling-ball viscometry. J. Fluid Mech..

[22] Goldman A.J., Cox R.G., Brenner H. (1967). Slow viscous motion of a sphere parallel to a plane wall—I Motion through a quiescent fluid. Chem. Eng. Sci..

[23] Joseph D., Nelson J., Hu H., Liu Y. (1992). Competition between inertial pressures and normal stresses in the flow induced anisotropy of solid particles. Theor. Appl. Rheol..

[24] HARRISON G.M., LAWSON N.J., Boger D. (2001). The measurement of the flow around a sphere settling in a rectangular box using 3-dimensional particle image velocimetry. Chem. Eng. Commun..

[25] Harrison G.M., Tatum J.A., Lawson N.J. (2004). A Study of the Sedimentation of a Sphere Near a Vertical Wall Using Three-Dimensional PIV. Proceedings of the Heat Transfer Summer Conference.

[26] Tatum J.A., Finnis M.V., Lawson N.J., Harrison G.M. (2005). 3-D particle image velocimetry of the flow field around a sphere sedimenting near a wall: Part 2. Effects of distance from the wall. J. Non-Newton. Fluid Mech..

[27] Yang S., Tu C., Dai M., Ge X., Xu R., Gao X., Bao F. (2021). Sedimentation of Two Side-by-Side Heavy Particles of Different Density in a Shear-Thinning Fluid with Viscoelastic Properties. Appl. Sci..

[28] Lockyer M., Davies J., Jones T. (1980). The importance of rheology in the determination of the carrying capacity of oil-drilling fluids. Rheology.

[29] Reynolds P., Jones T. (1989). An experimental study of the settling velocities of single particles in non-Newtonian fluids. Int. J. Miner. Process..

[30] Acharya A., Mashelkar R., Ulbrecht J. (1976). Flow of inelastic and viscoelastic fluids past a sphere. Rheol. Acta.

[31] Mansoor M.M., Vakarelski I.U., Marston J., Truscott T.T., Thoroddsen S.T. (2017). Stable–streamlined and helical cavities following the impact of Leidenfrost spheres. J. Fluid Mech..

[32] Vakarelski I.U., Jetly A., Thoroddsen S.T. (2019). Stable-streamlined cavities following the impact of non-superhydrophobic spheres on water. Soft Matter.

[33] Takemura F. (2004). Migration velocities of spherical solid particles near a vertical wall for Reynolds number from 0.1 to 5. Phys. Fluids.

[34] Jeong H., Park H. (2015). Near-wall rising behaviour of a deformable bubble at high Reynolds number. J. Fluid Mech..

[35] Małysa K., Van De Ven T. (1986). Rotational and translational motion of a sphere parallel to a wall. Int. J. Multiph. Flow.

[36] Takemura F., Magnaudet J. (2003). The transverse force on clean and contaminated bubbles rising near a vertical wall at moderate Reynolds number. J. Fluid Mech..

[37] Luo K., Wang Z., Fan J., Cen K. (2007). Full-scale solutions to particle-laden flows: Multidirect forcing and immersed boundary method. Phys. Rev. E.

[38] Luo K., Wei A., Wang Z., Fan J. (2013). Fully-resolved DNS study of rotation behaviors of one and two particles settling near a vertical wall. Powder Technol..

[39] Yao C., Zhang J., Xue Z., Yu K., Yu X., Yang X., Qu Q., Gan W., Wang J., Jiang L. (2021). Bioinspired cavity regulation on superhydrophobic spheres for drag reduction in an aqueous medium. ACS Appl. Mater. Interfaces.

[40] Veldhuis C.H.J. (2007). Leonardo’s paradox: Path and shape instabilities of particles and bubbles. Ph.D. Thesis.

[41] Kim S.J., Kim H.N., Lee S.J., Sung H.J. (2020). A lubricant-infused slip surface for drag reduction. Phys. Fluids.

